# The Contributions of the Amino and Carboxy Terminal Domains of Flightin to the Biomechanical Properties of *Drosophila* Flight Muscle Thick Filaments

**DOI:** 10.3390/biology5020016

**Published:** 2016-04-27

**Authors:** Nathan S. Gasek, Lori R. Nyland, Jim O. Vigoreaux

**Affiliations:** 1 Department of Biology, University of Vermont, Burlington, VT 05405, USA; ngasek@uvm.edu (N.S.G.); lori.nyland@med.uvm.edu (L.R.N.); 2 Department of Molecular Physiology and Biophysics, University of Vermont, Burlington, VT 05405, USA

**Keywords:** flightin, *Drosophila melanogaster*, myosin, thick filament, persistence length, atomic force microscopy

## Abstract

Flightin is a myosin binding protein present in Pancrustacea. In *Drosophila*, flightin is expressed in the indirect flight muscles (IFM), where it is required for the flexural rigidity, structural integrity, and length determination of thick filaments. Comparison of flightin sequences from multiple *Drosophila* species revealed a tripartite organization indicative of three functional domains subject to different evolutionary constraints. We use atomic force microscopy to investigate the functional roles of the N-terminal domain and the C-terminal domain that show different patterns of sequence conservation. Thick filaments containing a C-terminal domain truncated flightin (*fln*^Δ*C44*^) are significantly shorter (2.68 ± 0.06 μm; *p* < 0.005) than thick filaments containing a full length flightin (*fln^+^*; 3.21 ± 0.05 μm) and thick filaments containing an N-terminal domain truncated flightin (*fln*^Δ*N62*^; 3.21 ± 0.06 μm). Persistence length was significantly reduced in *fln*^Δ*N62*^ (418 ± 72 μm; *p* < 0.005) compared to *fln^+^* (1386 ± 196μm) and *fln*^Δ*C44*^(1128 ± 193 μm). Statistical polymer chain analysis revealed that the C-terminal domain fulfills a secondary role in thick filament bending propensity. Our results indicate that the flightin amino and carboxy terminal domains make distinct contributions to thick filament biomechanics. We propose these distinct roles arise from the interplay between natural selection and sexual selection given IFM’s dual role in flight and courtship behaviors.

## 1. Introduction

Thick filaments play a fundamental role in defining the sarcomeric structure and contractile properties of muscle. The major constituent of thick filaments in most muscle types is myosin II, a highly conserved protein characterized by a globular motor domain responsible for the chemomechanical basis of contraction, and a coiled-coil tail domain responsible for polymerization into highly ordered filaments [1]. Additional species and muscle-type specific proteins confer thick filaments with distinct structural and functional characteristics tailored to the specific operational demands of the muscle as evidenced, for example, in the various forms of animal locomotion such as running, hopping, flying, swimming, and crawling [2]. The predominant role of muscle in these various forms of locomotion often obscures its other important functions (e.g., heat production, sound production, metabolic health) that rely on contractile mechanisms that are operationally distinct from those used for locomotion. How a common thin and thick filament-based sarcomere design has evolved to fulfill different functional roles remains largely unknown.

Insect flight muscle engenders complex aerial behaviors involved in predation, mating rituals, and territorial protection, among others [3,4]. Insect flight muscle also facilitates ground-based behaviors involved in thermogenesis, ventilation, courtship, and sound generation, among others [5,6]. Studies in *Drosophila* and other insects are beginning to shed light on the adaptive mechanisms by which muscle fulfills multiple roles [7,8]. For example, mutations in the *Drosophila* myosin regulatory light chain manifest differently in flight and in the generation of the mating song, consistent with the hypothesis that the contractile based mechanisms underlying these two behaviors are different [9].

Flightin is a 20 kDa (182 amino acids) myosin coiled-coil binding protein required for the normal assembly and function of thick filaments in *Drosophila* flight muscles [10,11]. Thick filaments devoid of flightin assemble to abnormally long length, are structurally compromised, and are more compliant (*i.e.*, lower flexural rigidity) than their normal counterparts [12,13]. The absence of flightin renders the flight muscle inoperative due to a reduction in the viscoelastic response of fibers resulting in a significant loss of oscillatory work and power [14]. Additionally, sarcomere structure is compromised to the point it cannot withstand contractile forces [15]. Despite its critical role in flight muscle structure and function, little is known about the molecular properties of flightin and the mechanisms by which this important protein fulfills its functional roles.

**Figure 1:**
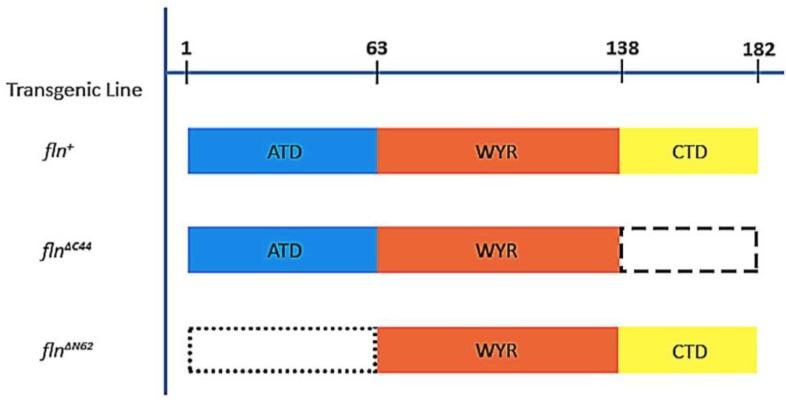
Representation of the three domains of flightin (fln) and their expression in three experimental strains. Top line numbers indicate amino acid position. The *fln*^Δ*C44*^ strain lacks the carboxy terminal domain (CTD) and *fln*^Δ*N62*^ lacks the amino terminal domain (ATD) of the protein. The *fln^+^* strain acts as a control transgenic line as it expresses a reintroduced full length flightin.

While flightin’s native structure is currently unknown, a comparison of its amino acid sequence amongst twelve *Drosophila* species revealed a tripartite organization indicative of three separate domains [16]. The region spanning the 84th to the 135th position (*D. melanogaster* numbering), referred to as the WYR domain for its conserved tryptophan (W), tyrosines (Y) and arginine (R), shows a high degree (>90%) of amino acid sequence identity among *Drosophila* and represents the only conserved flightin domain throughout the clade Pancrustacea [17]. The amino terminal residues 1 through 65 are highly variable and present a low degree (<20%) of sequence conservation. Compared to these two regions, the carboxy terminal region (amino acid residues spanning positions 137 through 182) shows intermediate conservation (60% identity) [16]. To establish if these regions that differ in amino acid sequence conservation represent independent protein domains, we created transgenic flies that express flightin devoid of the amino terminal region (*fln*^Δ^*^N62^*) and the carboxy terminal region (*fln*^Δ^*^C44^*) (Figure 1) [16,18]. Both forms of truncated flightin are incorporated into thick filaments and partially restore flight muscle functionality in *fln*^0^, a mutant strain that does not express flightin [12]. The extent to which flight muscle function is restored in *fln*^Δ^*^C44^* differs markedly from that in *fln*^Δ^*^N62^*, evidence in support of the hypothesis that the N-terminal region and the C-terminal region are protein domains with distinct functions.

This study was conducted to examine how the flightin N-terminal domain and the C-terminal domain contribute to the biomechanical properties of thick filaments. Using atomic force microscopy (AFM), the length, bending propensity, and persistence length of isolated, native thick filaments from *fln*^Δ^*^N62^* and *fln*^Δ^*^C44^* flies were compared to *fln^+^* control flies. Statistical polymer chain analysis revealed that each domain makes distinct contributions to thick filament biomechanics.

## 2. Materials and Methods

### 2.1. Drosophila Stocks

The generation of transgenic *fln^+^*, *fln*^Δ^*^N62^*, and *fln*^Δ^*^C44^* *Drosophila melanogaster* has been previously described [16,18,19]. All fly stocks were raised in vials of yeast agar food at 22 °C, constant humidity, and on a 12:12 light:dark cycle. Vials were cleared of adults and newly eclosed females collected daily for dissection, ensuring that all flies were between one and three days old.

### 2.2. Solutions

Rigor solution (pCa 4.5) consisted of 200 mM ionic strength, pH 7.0: 149.2 mM sodium methanesulfonate, 5 mM ethylene glycol bis(β-aminoethyl ether)*N*,*N*′-tetraacetic acid (EGTA), 4.97 mM CaCl_2_, 1.18 mM MgCl_2_, 8 mM Pi, 20 mM Bes-7.0 (*N*,*N*-bis(2-hydroxyethyl)-2-aminoethanesulfonic acid), 7.4 mM KOH, 2 mM DTT, and 1% (v/v) Protease Inhibitor Cocktail Set III (Merck, Darmstadt, Germany). Relaxing solution (pCa 8.0) consisted of 200 mM ionic strength, pH 7.1: 97.6 mM sodium methanesulfonate, 15 mM EGTA, 55 μM CaCl_2_, 6.88 mM MgCl_2_, 5.39 mM ATP, 8 mM Pi, 20 mM Bes-7.0, 2.4 mM KOH, 20 mM BDM (2,3-butanedione monoxime), and 1% (v/v) Protease Inhibitor Cocktail Set III (Merck). Dissecting solution consisted of rigor solution with 2% (v/v) Triton X-100 and 50% (v/v) glycerol. Imaging Solution (pCa 4.5) consisted of 150 mM ionic strength, pH 7.0: 114 mM sodium methanesulfonate, 5 mM EGTA, 5 mM CaCl_2_, 1 mM MgCl_2_, 20 mM Bes-7.0, 1 mM KOH, 2 mM DTT, and 1% (v/v) Protease Inhibitor Cocktail Set III (Merck). Calpain Solution contained 2 mg/mL calpain-1 from porcine erythrocytes (Merck), 20 mM imidazole HCl, 5 mM β-mercaptoethanol, 1 mM EDTA, 1 mM EGTA, and 30% glycerol, pH 6.8.

With the exception of the calpain solution and the protease inhibitors (Merck KGaA: Darmstadt, Germany), all reagents were obtained from Sigma (St. Louis, MO, USA).

### 2.3. IFM Thick Filament Isolation

For each transgenic line of flies, 21–30 female flies aged one to three days old were anaesthetized with CO_2_ and transferred to a dissection plate. The head, wings, abdomen, and legs were removed, leaving thoraces that were then transferred into a dissection disk containing iced dissecting solution with fresh protease inhibitors. Thoraces were then split in half and transferred to a dish of fresh dissecting solution. This dish was covered and incubated overnight at −20 °C. IFM fibers were then dissected from the half thoraces and transferred to a chilled dish with fresh dissecting solution. Next, the isolated IFM fibers were transferred to a 1.5 mL centrifuge tube with rigor solution and sheared once with a 20 G needle, followed by centrifugation at 200 rpm and 4 °C for 4 min. The supernatant was decanted, and fresh rigor solution was added. This wash sequence was repeated two additional times with centrifugations at 400 rpm and 600 rpm. After the final wash, all but 15 μL of the rigor supernatant was removed and 20 μL of 2 mg/mL calpain solution was added. Calpain activation was achieved by adding 40 μL of 0.1 M CaCl_2_. This solution was placed on a rocking tray at 22 °C for 35 min followed by an additional 5 min at room temperature. Sixty μl of the calpain and CaCl_2_ solution added was removed from the fibers and digestion was then completely stopped by the addition of 400 μL of relaxing solution containing fresh protease inhibitors. The fibers were then sheared 7 times with a 1 mL syringe fitted with a 20 G needle, producing a transparent filament suspension. This suspension was then centrifuged for 4 min at 2000× *g* and the supernatant was separated from the pellet for AFM imaging.

### 2.4. Atomic Force Microscopy Imaging

An 80-μL thick filament sample was allowed to incubate on a freshly cleaved mica substrate for 10 min after which 70 μL of excess solution was removed. Approximately 70 μL of imaging solution lacking protease inhibitors was then added to the prepared mica disk. Images were produced using an MFP3D Bioscope AFM (Oxford Instruments/Asylum Research, Santa Barbara, CA, USA) in tapping mode. Images were recorded at scan sizes of 5 or 6 μm and 512 × 512 pixels, providing images with pixel resolutions of 9.8 and 11.7 nm, respectively. The AFM probes used were the Budget Sensors (Innovatie Solutions Bulgaria Ltd, Sofia, Bulgaria) SiNi-30 short, silicone nitride tips.

### 2.5. Thick Filament Analysis

Images generated by the AFM were analyzed as described in a prior study [20]. Briefly, images were first processed in the proprietary format of the AFM via IgorPro (WaveMetrics Inc., Portland, OR, USA) where the images were flattened, planefit, and smoothed twice with a median filter. Images were then exported to the AFM’s accessory program ArgyleLite (Oxford Instruments/Asylum Research), where they were converted into ASCII matrix files that preserved pixel intensity from the AFM topography to be later analyzed in MatLab (MathWorks, Natick, MA, USA). Using a custom program written in Matlab and LabView, filaments were digitally rotated, aligned horizontally and isolated via image masking. These digitally isolated filaments were then fit with points placed perpendicular to the filament contour according to a Gaussian distribution to generate a digital filament consisting of a series of (x, y) coordinates. This provided a means of accurately measuring thick filament contour lengths (s). Along the individual contours, end-to-end length (R), and the angle between tangent vectors at the end of each contour (θ) were recorded. Using these measurements, thick filament persistence length was calculated on both a pooled and per-filament basis in order to retain information about the intrinsic variability in the stiffness of filaments from each sample. The individual filament persistence length, referred to as the Specific Persistence Length (SPL), was estimated according to Equation (1):

(1) $$\langle R^{2}(s)\rangle =4\lambda s\left(1-\frac{2\lambda }{s}(1-e^{\frac{-s}{2\lambda }})\right)$$

where (R^2^(s)) is the mean squared end-to-end length and λ is the SPL. Group persistence length was calculated by averaging the individual persistence lengths at each contour and estimating the steady state value that was approached. Pixel limitation was determined by using completely straight digital “test filaments”, as described previously [13].

### 2.6. Statistical Analysis

All values are reported as mean ± SE. Filament lengths were compared to the control, flightin rescued line (*fln^+^*) using a *t*-test. The Mann-Whitney U test, which operates on nonnormal distributions, was used to determine significance in the differences between SPL values and control. An ANCOVA multiple comparisons test allowed variations in contour length to be accounted for in the assessment of differences between steady state persistence lengths.

## 3. Results

### 3.1. Thick Filament Length

**Figure 2:**
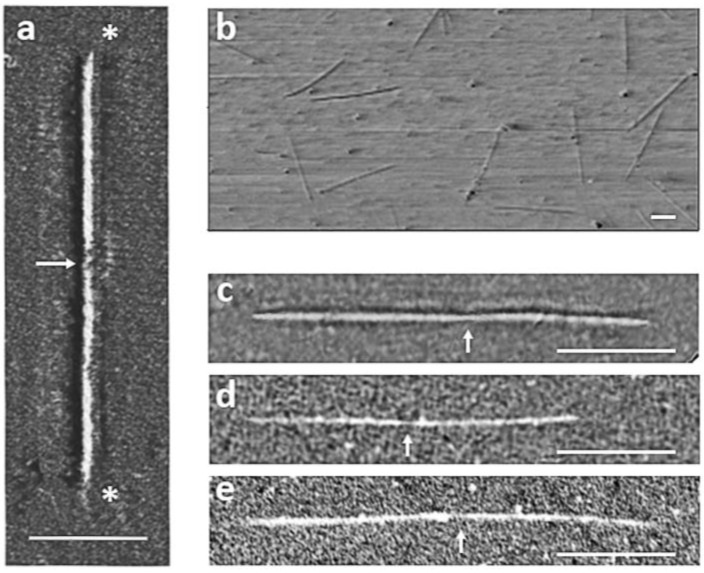
AFM images of isolated, native thick filaments on mica surface. (**a**) Most intact filaments (>67%) were identified by the presence of a bare zone near the halfway point (arrows) and tapered ends (*); (**b**) A wide field image reveals the relative homogeneity of isolated thick filaments. Representative thick filaments from *fln^+ ^*(**c**), *fln*^Δ*C44*^(**d**), and *fln*^Δ*N62 *^(**e**) were captured during independent imaging sessions. Differences in contrast correspond to different tapping force applied to optimize each imaging session. Scale bars = 1 μm.

**Figure 3:**
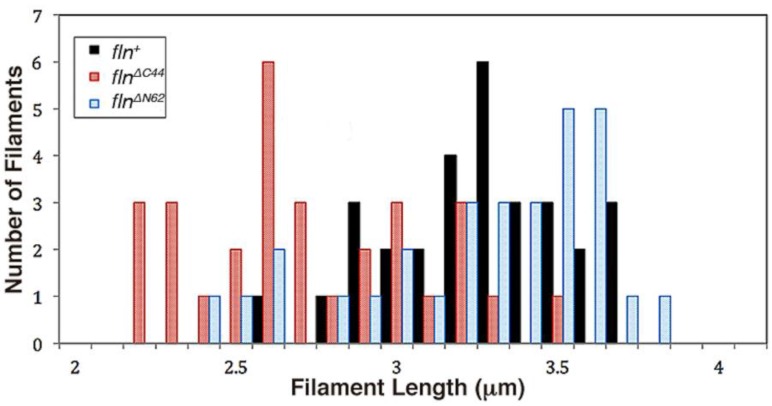
Indirect flight muscle thick filament length distribution amongst transgenic *Drosophila*. All filaments included fall within a 95% confidence interval of filament length.

AFM images of native thick filaments from the flightin rescued strain (*fln^+^*) were consistent with prior investigation of Oregon R wild type flies, showing filaments with characteristic bare zones and tapered ends (Figure 2) [13]. As an absence of these characteristics could be indicative of broken filaments, a 95% confidence interval of filament length was constructed to eliminate fragments from the sample. Using these criteria, 30 filaments were selected from each fly strain for analysis. The mean filament length of the control (*fln^+^*) group was 3.21 ± 0.05 μm. Filaments from *fln*^Δ^*^C44^* flies were significantly shorter (*p* < 0.005) than *fln^+^* with a mean filament length of 2.68 ± 0.06 μm (Figure 3). On the other hand, filaments from *fln*^Δ^*^N62^* flies showed no significant difference in filament length as compared to *fln^+^* control. In these flies, the mean thick filament length was 3.21 ± 0.06 μm (Table 1).

### 3.2. Thick Filament Bending Propensity

In order to investigate the contributions of flightin’s amino and carboxy terminal domains to thick filament mechanics, AFM images were analyzed in a custom MatLab script to determine filament bending propensity and persistence length [20]. The persistence length was calculated first on a per-filament basis, or specific persistence length (SPL) according to Equation (1). Filaments were excluded from analysis if they were not contained in the 95% confidence interval for length, or if their persistence length exceeded the value calculated for a digital “test filament” [13]. This test filament was a digitally constructed, completely straight filament that was subjected to the same analysis as the AFM images. Due to the pixel size limitations in the program, the persistence length of this test filament represented the upper bounds to the resolution of analysis due to pixel artifacting. There was no significant difference found between the SPL of *fln^+^* (1386 ± 196 μm) and *fln*^Δ^*^C44^* flies (1128 ± 193 μm). Filaments from the *fln*^Δ*N62*^ group were significantly less stiff than those from the *fln^+^*control strain (*p* < 0.005), with a mean SPL of 418 ± 72 μm (Table 1).

**Figure 4:**
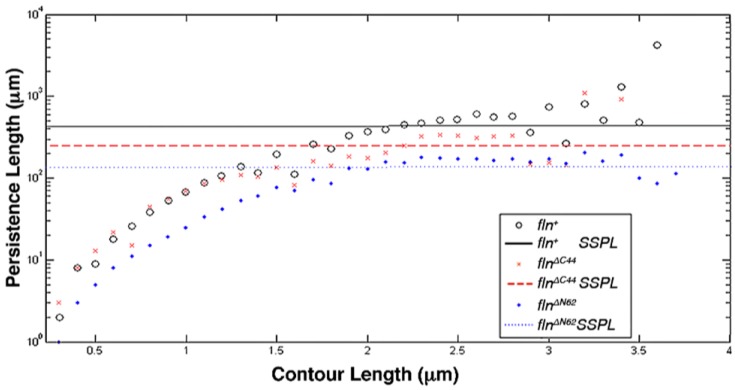
Persistence Lengths and Steady state persistence lengths (SSPL) were calculated for thick filaments from each population by determining the “per filament persistence length”, or specific persistence length (SPL), at each contour length. SPL values for filaments equilibrated onto 2-dimensional substrate were obtained according to Equation (1), and were then averaged at each 100 nm interval contour length. Horizontal lines indicate the line of best fit for persistence length at contours greater than 1.2 μm.

**Figure 5:**
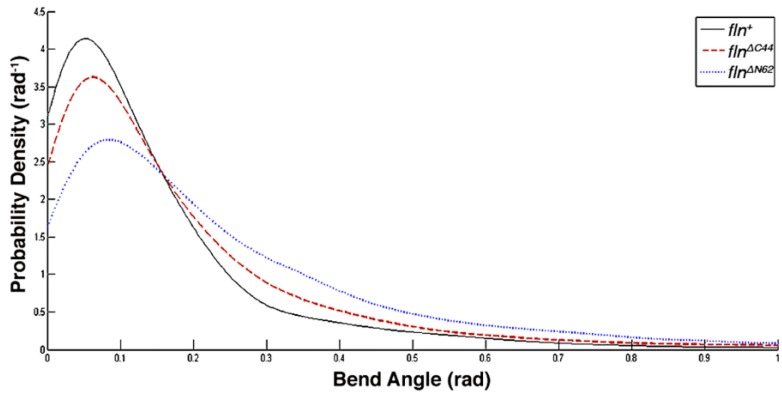
The von Misses distribution fit to the frequency of bend angles found in thick filaments from each transgenic line. Note that larger bend angles (>0.16 rad) are more frequently found in filaments lacking the amino terminal domain (blue dotted line) than in control (solid black line) and filaments lacking the carboxy terminal domain (red broken line). Similarly, smaller bend angles (<0.16 rad) are more frequently found in thick filaments from control flies and from flies expressing lacking the carboxy terminal domain.

In addition to the SPL, estimates of persistence length were calculated on a group basis. This measure, referred to as steady state persistence length (SSPL), has been suggested to be a better representation of filament compliance as it arises from a steady state calculation that ignores the pixilation artifacts presented by smaller contour lengths [13]. Using this approach, significant differences were found between the SSPL of all three groups (*p* < 0.05). SSPL was highest in *fln^+^* flies (431 μm), followed by the *fln*^Δ^*^C44^* group (268 μm). Consistent with the SPL measurements, thick filaments from *fln*^Δ*N62*^ were the most compliant with a SSPL of 146 μm (Table 1 and Figure 4). A similar trend is reflected in the von Misses probability density distribution of bend angle frequency (Figure 5). The *fln^+^* distribution is indicative of the stiffest filaments as large bend angles (θ > 0.16 rad) are the least frequent for this filament group, and small bend angles (θ < 0.16 rad) are the most frequently found in these filaments. The *fln*^Δ*N62*^ filament group follows the opposite trend as it displays the highest probability density of large bend angles and the lowest probability density of small bend angles. As with the persistence length measurement, the *fln*^Δ^*^C44^* flies presented an intermediate degree of compliance as their large and small bend angle distributions were between the extremes of the other two groups.

**Figure 6:**
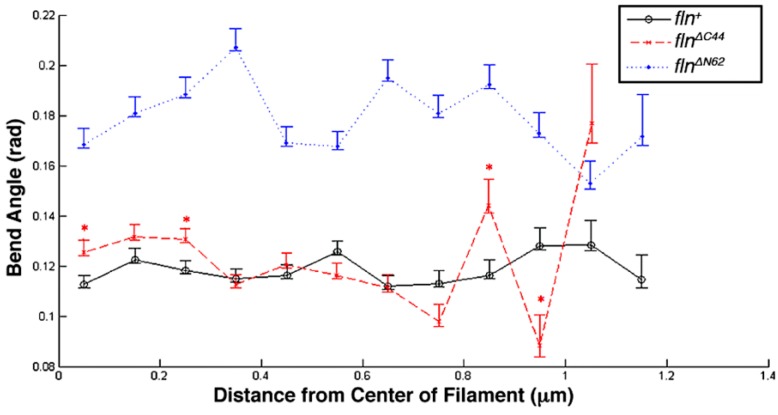
Mean bend angles at distances from the center of each population of thick filaments. Thick filament images were digitally folded in half, as was previously described in detail, and the mean bend angle at each distance from the center of the filament was plotted from a constant contour length of 1.2 μm, the half A-band length where flightin is distributed [20]. Bend angles at distances greater than 1.2 μm remain large, as previously noted [13]. Bend angles were significantly larger (*p* > 0.05) between the amino terminal deletion and control line at all distances. Significant differences between the carboxy terminal deletion and control are indicated with an asterisk. Variation in bend angles increases at filament tips where there are fewer data tracking points available. This is especially noticeable in the carboxy terminal deletion as the filaments were shorter and had fewer representative points at the furthest distances from the center of the filament.

To gain further insight into the effect of the flightin domain deletions on thick filament flexural rigidity, the bending propensity was examined along the length of the filament. For reasons previously described, filaments were digitally folded in half lengthwise and the mean bend angle along each half filament was plotted at intervals of 100 nm [20]. Using a contour length of 1.2 μm, the half A-band width that contains flightin, it was found that *fln^+^* thick filaments maintain a consistently high degree of stiffness throughout this region (Figure 6). While the *fln*^Δ^*^C44^* filament stiffness is comparable to the *fln^+^* filament stiffness, *fln*^Δ^*^C44^*filaments demonstrate significantly higher bending propensity at 0.2 and 0.4 μm, closer to the bare zone, and at 0.8 μm, approaching the filament tip. Though the mean bend angle was found to be lower in *fln*^Δ^*^C44^* *vs.* *fln^+^* at 0.9 μm, this trend was previously observed and attributed to increased error resulting from fewer data tracking points at the extreme ends of thick filaments [13]. Consistent with the other models of filament stiffness, *fln*^Δ*N62*^ filaments had significantly larger bend angles throughout the length of the filament as compared to *fln^+^*.

## 4. Discussion

The results presented here demonstrate that the amino terminal domain and the carboxy terminal domain of flightin make distinct contributions to the biomechanical properties of thick filaments, providing further insight into how flightin influences insect flight muscle functionality. Previously, we had shown that reintroduction of the full length flightin gene into a flightin null (*fln^0^*) strain (*i.e.*, the *fln^+^*control strain used here) renews the structural integrity and function of the flight muscle to nearly wild-type levels [19]. The extent of this phenotype rescue is further manifested here: the flightin rescue *fln^+^*flies and the previously characterized wild type OR flies contain thick filaments of comparable lengths (3.21 ± 0.05 μm and 3.20 ± 0.04 μm) and specific persistence lengths (1386 ± 196 μm and 1742 ± 266 μm) [13]. In contrast, the SSPL and mean bend angle values of thick filaments from *fln^+^* flies differed from those of OR flies reported previously [13]. While this, in part, can be attributed to genetic differences between the wild-type OR strain and the transgenic *fln^+^* stock, the different imaging substrate used in this study (mica) and the prior study (HOPG) is also a contributing factor. For this study a transparent mica substrate was used to take advantage of the AFM’s inverse optical scope during imaging. The difference in transparency, however, is also accompanied by a change in surface charge distribution. In contrast to the nonpolar and hydrophobic surface of HOPG, mica has a negative charge. As the thick filament is also highly negative in charge, repulsive forces influence the typical Brownian motion of thick filaments and reduce equilibration onto the substrate, as was shown with intermediate filaments [21]. In light of this, it is more appropriate to refer to the extrapolated filament stiffness indices as “apparent” persistence lengths, due to deviations in filament equilibration [20].

Our results indicate the amino terminal domain exerts greater influence on filament stiffness than the carboxy terminal domain, while the latter has a greater impact on filament length. This division of labor is reflected in the amino acid sequence. The carboxy terminal domain sequence is more conserved than the amino terminal domain sequence, consistent with its role in defining a fundamental characteristic of thick filaments. While information on how the length of thick filaments determines muscle functionality is incomplete, it is evident from the remarkable consistency in filament length within a sarcomere that mechanisms involved in length determination are strongly selected for. A role in defining thick filament length provides an explanation for our prior observation that the carboxy terminal domain sequence is strongly conserved within a taxon, but decays rapidly between taxa [17]. Among invertebrate muscles, thick filaments vary considerably in length (~1.5 to 50 μm), compared to their relatively narrow length range (~1.6 μm) in vertebrate skeletal muscle [1,22]. The exclusive presence of flightin in Pancrustacea, by far the most speciose clade, may afford these organisms greater latitude in tinkering with the common sarcomere design, and hence muscle functionality, through differences in thick filament length and architecture. A comparison of thick filament lengths between *fln*^Δ*C44*^ and *fln^+^* flies (2.68 ± 0.06 μm and 3.21 ± 0.05 μm, respectively) is imitative of the reduction in sarcomere length found between these transgenic strains (3.12 ± 0.02 μm for *fln*^Δ*C44*^ *vs.* 3.42 ± 0.04 μm for *fln^+^*) [16]. This suggests that factors influencing thick filament length may also impose constraints on higher-level sarcomere structure.

Of the three transgenic fly lines studied here, only *fln*^Δ*C44*^ flies are incapable of beating their wings [16]. Transitively, the results presented here showing a significant decrease in filament length and, to an extent, flexural rigidity provides a potential mechanism for the reduced power output observed in *fln*^Δ*C44*^, as shorter filaments have fewer molecular motors. This is consistent with the reduction observed in modeled parameters B and C, indicative of a decrease in the number of strongly bound cross-bridges during active contraction. Additionally, deletion of the carboxy terminal domain affects myofilament lattice organization and decreases cross-bridge cycling kinetics by reducing the rate of cross-bridge recruitment [16]. Furthermore, sarcomeres in *fln*^Δ*C44*^ flies have characteristic defects in M-line structure, suggesting that the carboxy terminal domain may facilitate a stabilizing interaction with an M-line protein such as miniparamyosin [23]. As miniparamyosin is also distributed at the thick filament tips, an interaction with the flightin carboxy terminal domain may also explain the greater variation in bend angle profile at the terminal ends of *fln*^Δ*C44*^ thick filaments [23]. In terms of SPL, there was no significant difference between the thick filaments found in the *fln^+^* and *fln*^Δ*C44*^ flies. This is reflected in the mechanical performance of single skinned fibers as there is a consistency between the elastic modulus of *fln^+^* and *fln*^Δ*C44*^ fibers under relaxing conditions [16]. In summary, the direct effect of the carboxy terminal domain on thick filament length may underlie the myofilament lattice disorder and compromised contractile kinetics with consequent loss of flight in *fln*^Δ*C44*^ flies.

The amino terminal domain is the least conserved region in flightin. The marked influence of this region on filament stiffness provides a possible explanation for its lack of sequence conservation. Thick filament stiffness influences fiber stiffness, which in turn defines muscle functional outcomes [22]. Altering the amino acid sequence of the N-terminal domain may be a mechanism for fine-tuning filament stiffness without compromising basic contractile function. This is evident inasmuch as *fln*^Δ*N62*^ flies are flight competent and have normal wing beat frequency [18]. The flight muscle is responsible for generating the wing beats required for flight and for production of the mating song, two key behaviors with distinct muscle power requirements and subject to different evolutionary constraints. Among Drosophilids, mating songs are species-specific and have evolved in response to sexual selection and contributed to speciation [24,25]. As genes under sexual selection tend to evolve at a faster rate, we speculate that the amino terminal domain of flightin contributes to species-specific song attributes via its role in thick filament stiffness.

## 5. Conclusions

In conclusion, the assigned functions to the amino and carboxy terminal domains are consistent with their observed levels of evolutionary sequence conservation. Collectively, this study affirms the critical role of flightin in the mechanics of insect flight and possibly mating song production. Further studies will be needed to identify the amino terminal and carboxy terminal domain features that underlie their functional roles.

## Figures and Tables

**Table 1:** Length parameters of isolated, native thick filaments from *Drosophila* IFM.

Transgenic Line	Filament Length (μm)	Specific Persistence Length (μm)	Steady State Persistence Length (μm)
*fln^+^* (N = 30)	3.21 ± 0.05	1386 ± 196	431
*fln*^Δ*C44*^** (N = 27)	2.68 ± 0.06 ^a^	1128 ± 193	268 ^c^
*fln*^Δ*N62*^** (N = 21)	3.21 ± 0.06	418 ± 72 ^b^	146 ^c^

Values are expressed as mean ± SEM. For each line, 30 thick filaments were sampled from N# of flies. ^a^ significant difference from *fln^+^*, *p* < 0.005 via *t*-test; ^b^ significant difference from *fln^+^*, *p* < 0.005 via Mann-Whitney U test; ^c^ significant difference from *fln^+^*, *p* < 0.05 via ANCOVA.
